# Long non-coding RNA CTSLP8 mediates ovarian cancer progression and chemotherapy resistance by modulating cellular glycolysis and regulating c-Myc expression through PKM2

**DOI:** 10.1007/s10565-021-09650-9

**Published:** 2021-09-12

**Authors:** Xiaoduan Li, Yi Zhang, Xinjing Wang, Feikai Lin, Xi Cheng, Ziliang Wang, Xipeng Wang

**Affiliations:** 1 Department of Obstetrics and Gynecology, XinHua Hospital Affiliated to Shanghai JiaoTong University School of Medicine, Shanghai, 200092 China; 2 The Reproductive Center, XinHua Hospital Affiliated to Shanghai JiaoTong University School of Medicine, Shanghai, 200092 China; 3 Department of Gynecological Oncology, Fudan University Shanghai Cancer Center, Shanghai, 200032 China

**Keywords:** Ovarian cancer, CTSLP8, Glycolysis, Resistance, PKM2, c-Myc

## Abstract

**Purpose:**

Long non-coding RNAs (lncRNAs) play vital roles in tumor progression and resistance. Ovarian cancer (OC), a common gynecological cancer, is associated with poor prognosis as it can progress to peritoneal metastasis and develop resistance to chemotherapy. This study aimed to examine the role of lncRNAs in the development of chemotherapy resistance in OC.

**Methods:**

The clinical samples were divided into chemotherapy-sensitive and chemotherapy-resistant groups based on the chemotherapy response at follow-up. The glycolysis levels in the two groups were analyzed using positron emission tomography/computed tomography (PET/CT) scanning and immunohistochemistry. GEO dataset analysis revealed the expression of CTSLP8 in chemotherapy-resistant patients with OC. Two pairs of normal and diamminodichloroplatinum (DDP)-resistant cells were transfected with CTSLP8 overexpression and knockdown constructs to examine the functions of CTSLP8 in the OC cells and elucidate the underlying mechanisms. The in vivo effect of CTSLP8 overexpression and knockdown on the chemotherapy response of tumors was examined using a mouse subcutaneous tumor model. The tissue chips were subjected to fluorescence in situ hybridization and immunohistochemical (IHC) staining to examine the correlation among CTSLP8 expression, DDP resistance, and prognosis in OC.

**Results:**

The dataset analysis demonstrated that CTSLP8 was upregulated in chemotherapy-resistant tumor tissues. CTSLP8 promoted the proliferation and development of DDP resistance in the OC cells. Moreover, CTSLP8 promoted c-Myc expression by facilitating the binding of PKM2 to the promoter region of c-Myc, thereby upregulating glycolysis. The analysis of tissue chips revealed that the upregulation of CTSLP8 was associated with the development of DDP resistance and poor prognosis in patients with OC.

**Conclusions:**

These findings indicate that CTSLP8 forms a complex with PKM2 to regulate c-Myc, and this action results in the upregulation of cellular glycolysis, consequently promoting OC progression and development of chemotherapy resistance.

**Headlights:**

1. CTSLP8 was upregulated in the chemotherapy-resistant tumor tissues.

2. CTSLP8 promoted the proliferation and cisplatin resistance in the OC cells.

3. CTSLP8 promoted glycolysis by facilitating the binding of PKM2 to the promoter region of c-Myc.

4. Inhibition of CTSLP8 or the combination of c-Myc inhibitors with cisplatin were potential therapeutic strategies for chemotherapy-resistant of OC.

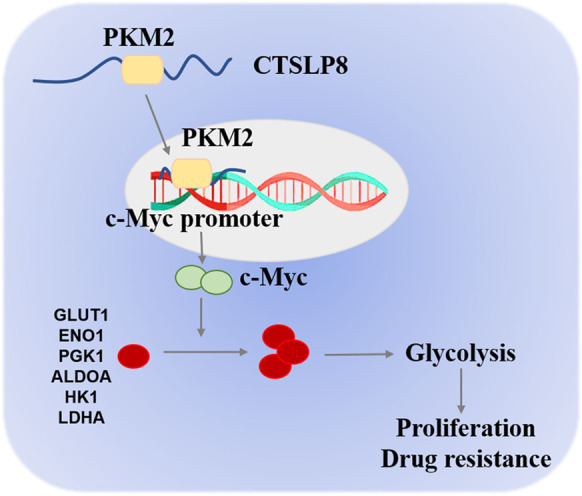

**Supplementary Information:**

The online version contains supplementary material available at 10.1007/s10565-021-09650-9.

## Introduction

LncRNAs are non-coding RNA characterized by the size of more than 200 nucleotides (Martens-Uzunova et al. 2014). Previously, lncRNAs were considered intracellular junk as they do not code for proteins. However, studies in the last decade have demonstrated the role of lncRNAs in many processes, such as cell proliferation, differentiation, and so on (Peng et al. 2017). In particular, lncRNAs are involved in transcriptional silencing and activation, chromosome modification, and nuclear transport (Lin and Yang 2018). Furthermore, the expression of lncRNAs is dysregulated in tumors, which results in the generation of signals that promote malignant transformation. Hence, lncRNAs are potential therapeutic targets for cancer (Schmitt and Chang 2016).

LncRNAs promote the progression of malignant tumors by interacting with DNA, proteins, RNA, and so on. Various studies examining the regulatory mechanism of lncRNAs have mostly focused on the competing endogenous RNA (ceRNA) mechanism and the correlation among lncRNA, miRNA, and mRNA. However, lncRNAs can also regulate transcription factors or transcription co-activators and consequently modulate the expression of target molecules. Moreover, the direct interaction between lncRNAs and proteins has piqued the interest of the scientific community (Wong et al. 2018). NEAT1 mediates the interaction between forkhead transcription factor (FOXN3) and the SIN3A complex, and the complex is involved in mediating EMT and metastasis of breast cancer (Li et al. 2017). The nuclear factor κB-interacting lncRNA (NKILA) can bind to NF-κB/IKB to form a stable complex, which suppresses breast cancer metastasis by inhibiting the IKB phosphorylation and NF-κB activation (Liu et al. 2015). LncRNA-Cox2, a responsive lncRNA induced by tumor necrosis factor-α, enhances the transcription of *Il12B*, and it is important in the regulation of intestinal epithelial inflammatory responses (Tong et al. 2016). The expression of lncRNA PVT1 in the hepatocellular carcinoma (HCC) tissues is increased when compared with that in the HBV-negative tissues. PVT1 promotes c-Myc expression and HCC progression by altering histone methylation at the c-Myc promoter (Jiang et al. 2020). The deficiency of FOXO-induced long non-coding RNA 1 (FILNC1) promotes glycolysis through the upregulation of c-Myc (Xiao et al. 2017). These findings demonstrate that in addition to the ceRNA mechanism, lncRNAs can modulate functional proteins through direct interaction.

Ovarian cancer (OC) is one of the most fatal malignancies of the reproductive system, and approximately 75% of cases are diagnosed at an advanced stage (Siegel et al. 2020). Additionally, OC can progress to peritoneal metastasis and develop chemotherapy resistance. Thus, the 5-year survival rate of OC patients has not improved (Lheureux et al. 2019). Platinum (diamminodichloroplatinum, cisplatin, DDP)-based and paclitaxel (TX)-based chemotherapeutics are the first-line treatment for patients with OC. The development of DDP resistance in OC cells decreases the efficacy of chemotherapeutics (Agarwal and Kaye 2003). Various studies have reported the role of lncRNAs at all stages of OC development (Tripathi et al. 2018). For example, lncRNA MAP3K20 antisense RNA, which is upregulated in the OC tissues, modulates the EMT process by competitively inhibiting miR-375 (Yan et al. 2018). LINC00092 has been reported to promote cancer-associated fibroblast-mediated OC progression and metastasis by regulating the glycolytic cycle of the OC cells (Zhao et al. 2017). The experimental studies have confirmed that silencing or functional inhibition of some specific lncRNAs is a potential therapeutic strategy for patients with OC.

There are limited studies that have examined the mechanisms underlying chemotherapy resistance. Recent studies have demonstrated that lncRNAs regulate the development of drug resistance in cancer (Wei et al. 2019). The lncRNA nuclear paraspeckle assembly transcript 1 (NEAT1), an estrogen-inducible lncRNA, is reported to be upregulated in TX-resistant endometrial cancer. The inhibition of NEAT1 expression enhances the reactivity of endometrial cancer cells to TX (Dong et al. 2019). LncRNA H19 promotes the activation of autophagy, leading to tamoxifen resistance in breast cancer cells. Thus, the knockdown of H19 reverses tamoxifen resistance in cells (Wang et al. 2019). However, some lncRNAs exert opposite effects. The expression of lncRNA GAS5 decreases in OC tissues and cell lines, especially in DDP-resistant cell lines. Moreover, overexpression of GAS5 enhances the sensitivity of OC cells to DDP (Long et al. 2019). In this study, analysis of GEO dataset showed the expression of lncRNA CTSLP8 was increased in chemotherapy-resistant OC tissues. Additionally, the correlation between chemotherapy resistance and tumor glycolysis was examined.

Cancer cells are characterized by upregulated glycolysis, which is called the Warburg effect. Upregulated glycolysis is associated with various characteristics of cancer aggressiveness, including proliferation, immune response, and drug resistance (Icard et al. 2018). The oncogenic lncRNA differentiation antagonizing non-coding RNA regulates miR-125b-5p expression to change the expression of HK2, which is related to the upregulation of glycolysis, eventually leading to the development of DDP resistance in colon cancer (Shi et al. 2020). The lncRNA urothelial carcinoma-associated 1 (UCA1) promotes chemoresistance in pediatric acute myeloid leukemia by upregulating glycolysis through the miR-125a/HK2 pathway. The knockdown of UCA1 or 2-deoxy-glucose (2-DG)-mediated glycolysis inhibition attenuates UCA1-induced chemoresistance (Zhang et al. 2018). Moreover, some lncRNAs exert adverse effects on metabolism and drug resistance. For example, HIF-1α-stabilizing lncRNA (HISLA) inhibits the degradation of HIF-1α and consequently promotes aerobic glycolysis. The inhibition of HISLA suppresses the glycolytic pathway and the development of chemoresistance in breast cancer (Chen et al. 2019). These studies indicate that some lncRNAs aberrantly activate glycolysis, which alters the drug resistance status of cancer cells.

There are limited studies on the correlation between CTSLP8 expression and DDP resistance in OC. This study aimed to elucidate the mechanism underlying CTSLP8-mediated OC proliferation, glycolysis, and drug resistance. The findings of this study indicated that CTSLP8 can directly bind PKM2 and promote the binding of PKM2 to the promoter region of c-Myc. Moreover, CTSLP8 knockdown affected the drug resistance status of OC cells. Thus, CTSLP8 may serve as a therapeutic target for OC.

## Materials and methods

### Patients

In total, 81 OC tissues were collected from patients with OC at XinHua Hospital between 2018 and 2019. All patients were admitted for the first time and did not undergo chemotherapy prior to sampling. The International Federation of Gynecology and Obstetrics (FIGO) classification was used for pathological diagnosis, stage, and grade of patients with OC (Table 1). All participants were informed about the objectives and implications of the study before obtaining signed written informed consent. The study was permitted by the Institutional Review Board of XinHua Hospital (approval number. XHEC2018070).

**Table 1:** The clinicopathological characteristics of patients with ovarian cancer exhibiting varying expression levels of long non-coding RNA CTSLP8

Variables	*N*	CTSLP8 expression (mean ± SEM)	*p* value
Age			
≤ 50	25	16.45 ± 2.92	0.153
> 50	56	15.71 ± 2.43	
Clinical stage			
I + II	20	5.04 ± 4.47	< 0.001
III + IV	61	19.51 ± 1.85	
Histological subtype			
SOC	69	12.40 ± 2.95	0.105
NSOC	12	19.77 ± 5.36	
Histological grade			
Low	25	10.42 ± 4.11	0.001
High	56	19.71 ± 1.93	

*SOC* serous ovarian cancer; *NSOC* non-serous ovarian cancer

The tissue chips used for prognostic analysis comprised 124 tissues from patients with OC admitted to the Shanghai First Maternity and Infant Hospital with detailed prognostic information. The relevant sample information had been described in detail in a previous study (Zhou et al. 2018; Li et al. 2020).

### Analysis of TCGA and GEO datasets

The expression of CTSLP8 in OC tissues and other tumor tissues from TCGA dataset was normalized using variance stabilizing transformation. The CTSLP8 levels at different stages of tumors were comparatively analyzed, and the abbreviations for various tumors can be found in the supplementary data. The results obtained from the dataset GSE28646/GSE30161/GSE102073 were used for chemotherapy-resistant analysis, and those from the dataset GSE102073 were used for prognostic analysis. The best cutoff value of CTSLP8 expression was generated from the X-tile plot, and samples with CTSLP8 expression higher than the best cutoff were classified as the high expression group, while lower than the cutoff value were the low expression group. Then, the *p* value was calculated using log-rank test (Mantel-Cox). The tumors with enhanced levels of CA125 within 6 months of chemotherapy were defined as chemotherapy-resistant (chemo-resistant), while other tumors were defined as chemotherapy-sensitive (chemo-sensitive).

### Cell culture and transfection

SKOV3 and its platinum-resistant strain (SKOV3-DDP) were used in RPMI-1640 medium (Gibco; C11875500CP), and then 10% fetal bovine serum (FBS; Gibco; 10,099,141) and 1% penicillin–streptomycin solution (P/S; Gibco; 15,140,122) were added to prepare the complete medium. A2780 and its corresponding platinum-resistant strain (A2780-DDP) were cultured in high-glucose Dulbecco’s modified Eagle medium (DMEM, Gibco; C11995500BT) supplemented with 10% FBS and 1% P/S. HEK293T cells were cultured under the same conditions as those for A2780 cells. The cells were not cultured for more than 10 passages. Additionally, all cells were subjected to short tandem repeat analysis (STR) every 6 months. The last STR analysis was performed in May 2020.

To establish stable CTSLP8 overexpression or knockdown cell lines, the cells were transfected with CTSLP8 overexpression (P8-OE) or CTSLP8 knockdown (P8-KD) lentiviral constructs (designed by Genomeditech, China), respectively, following the instructions. The tumor cells were seeded in six-well plates at a confluency of approximately 50–60%. Then replace the culture medium with a fresh medium containing polybrene (6 µg/mL) with the appropriate concentration of lentivirus. Puromycin (2 µg/mL) was used for positive cell selection.

### Transfection of siRNA and plasmid

The siRNAs were transfected using the riboFECT CP transfection kit 166 T (RiboBio; C10511-05). The siRNAs against *PKM2* were synthesized by Guangzhou RiboBio Co. Ltd. The target cells were cultured in six-well plates till confluency of 70–80%. The siRNAs were mixed with the transfection reagent and riboFECT CP buffer as instructions. Then the cells were transfected with the siRNAs and negative control (NC), respectively. The knockdown efficiency of siRNAs was verified by examining the RNA and protein levels at 36 and 48 h, respectively.

The Promega transfection reagent FuGENE HD (Promega; E2311) was used for plasmid transfection. PKM2 overexpression, green fluorescent protein-tagged PKM2 (PKM2-GFP), and red fluorescent protein-tagged CTSLP8 (CTSLP8-RFP) plasmids were synthesized by Genomeditech (China). The protocols for plasmid transfection were similar to those used for siRNA transfection. The cells were planted in six-well plates till 70–80% confluency. Next, 2.0-µg plasmid was mixed with 5-µL transfection reagent, and then the mixture was added to 100-µL Opti-MEM (Thermo, 31,985,062) and incubated for 15 min. The cells were then transfected with the mixture for 6 h. For co-transfection, the cells were simultaneously transfected with the PKM2-GFP and CTSLP8-RFP plasmids. The transfection efficiency of the PKM2 overexpression plasmid was verified by examining the RNA and protein levels at 36 and 48 h, respectively. Furthermore, the transfection efficiency of the PKM2-GFP and CTSLP8-RFP plasmids was confirmed based on GFP and RFP fluorescence intensity at 24- to 36-h post-transfection.

### Immunohistochemical (IHC) staining of c-Myc and glycolytic enzymes

The IHC staining was used to examine the expression levels of c-Myc and glycolytic enzymes. The paraffin-embedded sections were incubated in a pressure cooker containing antigen retrieval buffer (pH 6.0) (Solarbio; G1202). Next, the slides were uniformly covered with 3% bovine serum albumin (Solarbio; A8020), and probed with the following primary antibodies prepared in a wet box at 4 °C overnight: anti-c-Myc (Servicebio; GB13076), anti-GLUT1 (Proteintech, 21,829–1-AP), anti-ENO1 (Proteintech, 11,204–1-AP), anti-PGK1 (Proteintech, 17,811–1-AP), anti-ALDOA (Proteintech, 11,217–1-AP), anti-HK1 (Proteintech, 19,662–1-AP), and anti-LDHA (Proteintech, 19,987–1-AP) antibodies. Next, the horseradish peroxidase (HRP)-labeled secondary antibody was used for 50 min. Then, the sections were then counterstained with hematoxylin.

The stained sections were scanned using Pannoramic MIDI and analyzed using Quant Center, which automatically identified all the strongly positive, moderately positive, weakly positive, and negative areas in the tissue sections. The H-scores were calculated as ∑ (percentage of intensity × intensity). The staining intensity was divided into three categories—strong, moderate, and weak—and the corresponding score was 3, 2, and 1, respectively.

### Western blotting

The expression of c-Myc and key glycolytic enzymes was also analyzed by western blotting. The proteins were isolated from cells with a density above 90% in a 10-cm disc. Two hundred microliter RIPA was used for lysing cells. The cell lysates were carefully scraped with a cell scraper and centrifuged. The protein content was quantified by BCA Kit (Thermo 23,227). To extract proteins from the tissues, the tissues were cut into small pieces, washed with PBS, collected in the centrifuge tube, and washed with PBS again. The tissue was lysed using 10 times the tissue volume of RIPA lysis buffer.

Total proteins were subjected electrophoresis using SDS gels. Then the 0.45-um polyvinylidene fluoride membrane (Millipore; IPVH00010) and 5% non-fat milk were used for blocking. The following primary antibodies were used to incubation: anti-c-Myc (Proteintech; 10,828–1-AP), anti-PKM2 (Proteintech; 15,822–1-AP), anti-actin (internal control) (CST; 4970), anti-GLUT1, anti-ENO1, anti-PGK1, anti-ALDOA, anti-HK1, and anti-LDHA antibodies. The protein signals were exposed by enhanced chemiluminescence solution (Millipore; WBKLS0500). Finally, the gray values of the bands were analyzed.

### qRT-PCR

Trizol was used for RNA extraction, and a reverse transcription kit (Takara; RR036A) was used for reverse-transcribed into cDNA. The cDNA was subjected to PCR using an RT-PCR kit (Takara; RR820A). The threshold cycle (Ct) and melting curves of the target genes were analyzed. The relative expression levels were calculated using the 2^−ΔΔCt^ method.

The primer sequences were as follows: *c-Myc*, 5′-GGCTCCTGGCAAAAGGTCA-3′ (forward) and 5′-CTGCGTAGTTGTGCTGATGT-3′; *PKM2*, 5′-ATGTCGAAGCCCCATAGTGAA-3′ (forward) and 5′-TGGGTGGTGAATCAATGTCCA-3′; CTSLP8, 5′-CCATCTCTGTTGCTGTTG-3′ (forward) and 5′-TCCTTCCTCATCACCATC-3′; β-actin, 5′-CATGTACGTTGCTATCCAGGC-3′ (forward) and 5′-CTCCTTAATGTCACGCACGAT-3′.

### CCK8 and colony formation assay

The suspension of trypsin-digested OC cells was inoculated in a 96-well plate (approximately 1200–1500 cells per well containing 200 µL of culture medium). The cells were treated with 8-mM 2-DG (MCE, HY-13966) daily. Day 0 was considered from 6 to 8 h after adherence. At 24-, 48-, 72-, 96-, and 120-h post-cell adherence, the supernatant was removed, 10-µL CCK8 solution (Beyotime; C0041) and 100-µL complete medium were added, and the cells were then incubated for 2 h at 37 °C. The absorbance was measured at 450 nm (BioTek; Synergy H4).

For the colony formation assay, the trypsin-digested OC cells were inoculated in a six-well plate (500 cells/well containing 2 mL of culture medium). The cells were observed every 3 days for the colony formation. The cells in the treatment group were treated with 8-mM 2-DG every day. The colony formation was observed after 10–15 days. The medium was removed, and then the cells were fixed and washed, then stained with crystal violet (Beyotime; C0121). Finally, remove crystal violet and collect images under a low-power microscope to count the number of clones.

### Evaluation of extracellular acidification rate (ECAR) and glycolysis analysis and metabolomic analysis

ECAR was measured using the Cell Mito Stress Test Kit (Agilent Technologies, 03,015–100). The trypsin-digested OC cells were inoculated in a 96-well Agilent Seahorse XF cell culture microplate (approximately 5000–6000 cells per well). The cells spread evenly throughout the well. ECAR was examined using a Seahorse XFe24 instrument at the Shanghai NanoBioscience Co. Ltd.

Glucose Uptake Assay Kit (Biovision, K676-100), Lactate Assay Kit (Biovision, K667-100), ATP Assay Kit (Promega, FF2000), and NADPH Assay Kit (AAT Bioquest; AAT-15272) were used to examine the products of glycolysis in the OC cells referring to the instructions.

An untargeted metabolomic analysis was performed using mass spectrometry, which involves the following steps: sample pretreatment, metabolite extraction, full scan detection in liquid chromatography-tandem mass spectrometry, data pretreatment, statistical analysis, and differential structure identification. Approximately 1 × 10^7^ cells from each sample were used with six replicates. The cells were fixed with 1-mL mixture (2methanol: 2acetonitrile: 1water). Metabolomic analysis and mass spectrometry were performed by Shanghai Applied Protein Technology.

### Determination of half-maximal inhibitory concentration (IC_50_) of cisplatin

The CCK8 assay was used to measure the IC_50_ of cisplatin in OC cell lines. The tumor cells were cultured in 96-well plates with a density above 90% (approximately 6000–8000 cells/well), and then the cells were treated with cisplatin (MCE, HY-17394) to the final concentrations of 0.2, 0.39, 0.78, 1.56, 3.13, 6.25, 12.5, 25, 50, and 100 µg/mL. And the log concentration was used in the IC_50_ figures. Each treatment group was comprised of three duplicate wells and the final reaction volume was 200 µL. To the NC group, an equal volume of DMSO was added. At 24- and 48-h post-treatment, the absorbance at 450 nm was measured as described in the CCK8 assay. The viability of the cells was calculated as follows.

$$\mathrm{Proliferation}\;\mathrm{inhibition}\;\mathrm{rate}=\left[{\mathrm{OD}}_{450}\;of\;the\;NC\;group-{OD}_{450\;}of\;the\;experimental\;group/{OD}_{450}\;of\;the\;NC\;group\right]\times100$$

### RNA pull-down, RNA-binding protein immunoprecipitation (RIP), and fluorescence resonance energy transfer (FRET)

CTSLP8 was synthesized using the overexpressed plasmid as a template and the sequence was verified by sequencing. The 3'-end of CTSLP8 was labeled using the Desthiobiotinylation kit (Thermo, 20,163) with T4 RNA ligase. The labeled CTSLP8 was used as a target for the protein-RNA interaction experiments, which were performed using the RNA–protein pull-down kit (Thermo, 20,164).

The RIP experiment used the Co-immunoprecipitation kit (Thermo, 26,149), except for the last step in which RNA in the eluent IgG/PKM2 was extracted directly using Trizol. The expression of CTSLP8 was examined by qRT-PCR.

For FRET experiments, the control group was transfected with PKM2-GFP plasmid (donor), while the experimental group was co-transfected with PKM2-GFP (donor) and CTSLP8-RFP plasmids (acceptor). The transfection efficiency was confirmed based on the fluorescence intensity of GFP/RFP. FRET experiments were performed at 36-h post-transfection using a TCS SMD FLCS Leica confocal microscope at the Shanghai Center for Plant Stress Biology.

### Dual-luciferase reporter and chromatin-immunoprecipitation (ChIP) assays

HEK293T cells were co-transfected with 1 µg of Promoter-NC + PKM2-NC/Promoter-NC + PKM2/Promoter-NC + CTSLP8/Promoter + PKM2-NC/Promoter + PKM2/Promoter + CTSLP8 luciferase and 20-ng Renilla plasmid. The ratio of luciferase activity to Renilla luciferase activity represents the relative expression level of luciferase. A dual-luciferase reporter kit (Promega; USA) was used to measure the luciferase activity after 48-h transfection.

For the ChIP experiments, 10^7^ cells were cross-linked with formaldehyde. The impurities were removed and the samples were fed with the antibody. After the input results were confirmed, the samples were transferred into two tubes. One aliquot of the sample was incubated with 10 µg of PKM2 antibody (Proteintech; 15,822–1-AP), while the other aliquot was incubated with 10 µg of IgG from the corresponding species. The samples were shaken and mixed overnight at 4 °C. The complexes were precipitated and purified. The precipitated DNA was analyzed using polymerase chain reaction to examine the fragment size. The primer sequences of the c-Myc promoter were divided into 10 regions. The following primers were used for ChIP analysis: F(1) CCCTTCTTGCTATTAAAAAAAAT and R(1) CTCGATCCCCCCGGGCTCAAA; F(2) GCTGCAGTGAGCTTTGATTGT and R(2) AACCGGTAATGGCAAACGTG; F(3) CTCCATAGGGTGATGTTCATT and R(3) AGTAAGTGTGCCCTCTACTG; F(4) TTACTTTCGCAAACCTGAACG and R(4) ACCACCTCCAAAAGAGAAAACAA; F(5) GGAGGGAGAGAAAAGTTTAC and R(5) GCCTTCCAGGCATTAATTTC; F(6) AGCCAAATTTTAATTAGCTCAAG and R(6) TATGGGAGGGGCAGGGGGTA; F(7) TTCTCCCGTCTAGCACCTTTGA and R(7) CAGCAGATACCGCCCCTCCT; F(8) CTTTGGCAGCAAATTGGGGGA and R(8) CCGCGCTTTGATCAAGAGTC; F(9) CCCTTTCCCCAGCCTTAGCG and R(9) GGTGGGGAGGAGACTCAGCC; F(10) TTCCCCACCCTCCCCACCCT and R(10) GATAAAGCCCCGAAAACCGG.

### Immunofluorescence analysis and fluorescent in situ hybridization (FISH)

The target cells were cultured in six-well plates until the density reached 50–60%. Next, the cells were fixed using paraformaldehyde, and incubated with an immunofluorescent blocking solution for 1 h. Then, the cells were dehydrated with a gradient alcohol series, soaked in wax, and embedded in paraffin. The sections were boiled in retrieval solution for 10–15 min and allowed to cool to room temperature.

The probe for CTSLP8 was designed to be Cy3 modified, and it was synthesized by Servicebio. The detailed sequence is as follows: 5'-Cy3-GGTTTTAACCTGATCCTTCACAGGACTCAT-3'. First, the probe for CTSLP8 was detected by matched FISH kit in Servicebio. The sections were then incubated with c-Myc antibody (Proteintech; 10,828–1-AP) and PKM2 antibody (Proteintech; 15,822–1-AP). Next, the secondary antibodies of different species were added, and the sections were incubated for 50 min. Finally, the images were captured by confocal microscope (Olympus; FV1000).

### In vivo experiment

Female athymic nude mice (*n* = 30; age, 6-week-old; bodyweight, 16–18 g) were supplied by Shanghai Slac Laboratory Animal Center and bred at specific pathogen-free (SPF) levels at the XinHua Hospital.

The mice were divided into five groups randomly. In both groups, the mice were subjected to bilateral subcutaneous injection with 100-µL PBS containing 5 × 10^6^ NC-transfected or P8-OE-transfected SKOV3 cells and 5 × 10^6^ NC-transfected or P8-KD-transfected SKOV3-DDP cells. Injecting different cells into the left and right sides of the same mouse can minimize the impact of individual differences.

A similar protocol was used to inject OC cells into the other three groups. The mice were subjected to bilateral subcutaneous injection with 100-µL PBS containing 5 × 10^6^ NC-transfected or P8-OE-transfected SKOV3 cells, 5 × 10^6^ NC-transfected or P8-KD-transfected SKOV3-DDP cells, or 5 × 10^6^ NC-transfected or P8-OE-transfected SKOV3 cells. At day 14 post-injection (2 weeks), the mice were intraperitoneally injected with cisplatin (4 mg/kg body weight), while IZCZ-3 (5 mg/kg body weight) (MCE; HY-111411) was injected subcutaneously at the base of the treated tumor. The drugs were administered twice a week for 6 weeks. A digital caliper was used to measure the tumor volume every 3 days. The tumor volume (V) was calculated by length (L) and width (W) using the formula: V = L × W^2^ × 0.72.

At week 6, the metabolism of all mice was evaluated by PET/CT scanning, and the maximum standard uptake value (SUV) was used to quantify the glucose uptake. Then subcutaneous tumors were dissected, photographed, and sectioned.

### Statistical analysis

Statistical analyses were performed using SPSS 20.0 software. The quantifiable values are presented as the mean ± SEM (standard error of mean). All in vitro experiments were repeated three times. The *p* values were calculated using the non-parametric, least significant difference, and Fisher’s exact tests. And overall survival (OS) was calculated using the Kaplan–Meier method. In figures, * denotes *p* < 0.05, ** means *p* < 0.01, and *** represents *p* < 0.001, while no significant difference is denoted by NS.

## Results

### Expression of CTSLP8 and the key glycolytic enzymes were upregulated in patients with chemotherapy-resistant OC

**Fig. 1:**
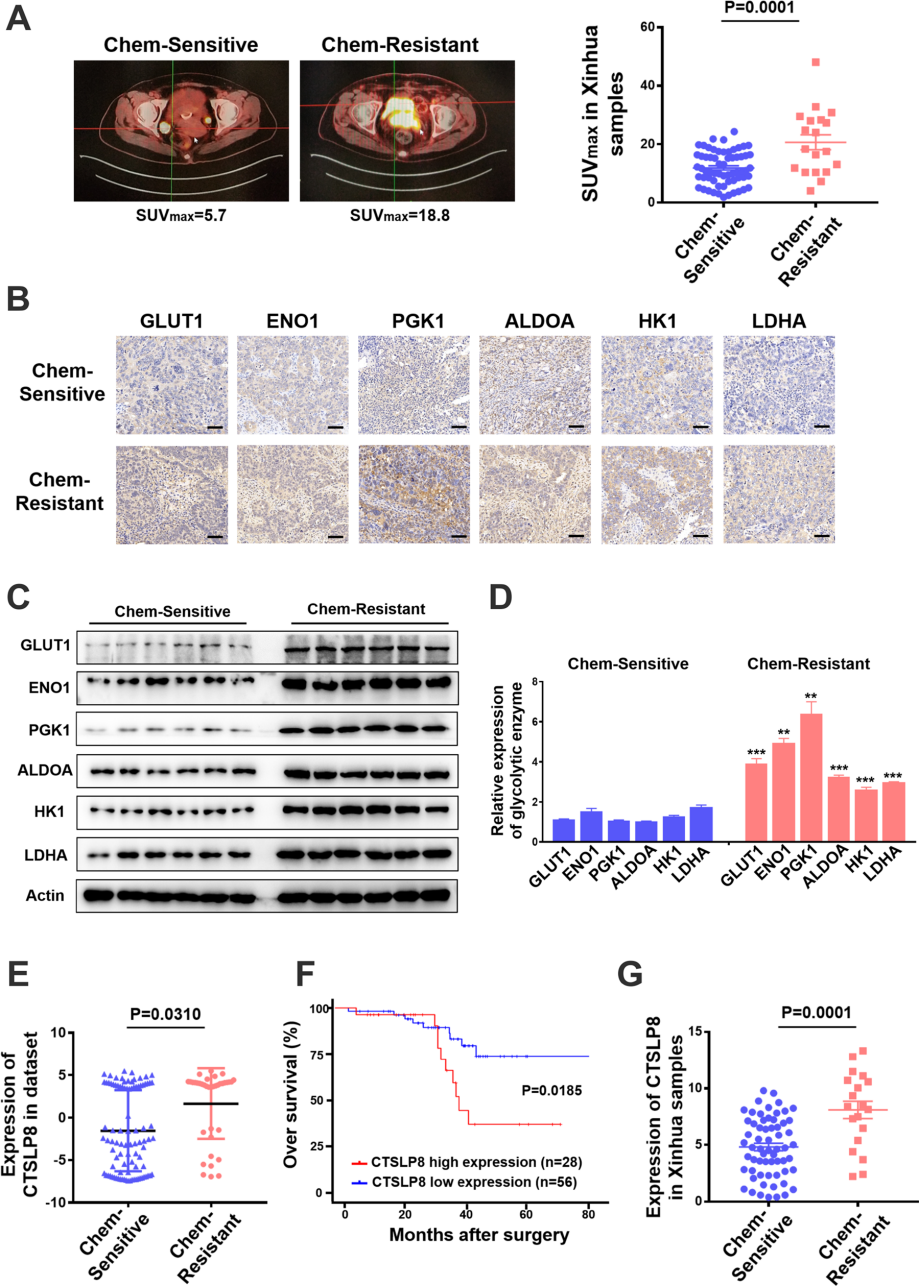
CTSLP8 and the expression of key glycolytic enzymes were upregulated in patients with chemotherapy-resistant OC. **A** Left, typical PET-CT images of chemo-resistant and chemo-sensitive patients. Right, the maximum SUV values of chemo-resistant (*n* = 19) and chemo-sensitive patients (*n* = 62) from Xinhua samples. **B** IHC staining of key glycolytic enzymes in the chemo-resistant and chemo-sensitive patients, including GLUT1, ENO1, PGK1, ALDOA, HK1, and LDHA. Scale bar, 50 µm. **C** Western blot analyzed the expression of key glycolytic enzymes in the chemo-resistant and chemo-sensitive patients. **D** The gray values of the key glycolytic enzymes in the chemo-resistant group and chemo-sensitive group. **E** The expression of CTSLP8 in patients with chemo-resistant and chemo-sensitive from GEO dataset. **F** The overall survival (OS) of OC patients in different CTSLP8 expression groups from dataset. **G** RT-PCR confirmed the expression level of CTSLP8 in 81 Xinhua tumor samples

In total, 81 patients with OC were screened at the Xinhua Hospital. The patients were classified as chemotherapy-resistant and chemotherapy-sensitive based on their postoperative chemotherapy response. The typical tumor images in the two groups were captured using PET/CT. The chemotherapy-resistant group exhibited a significantly higher SUV value than the chemotherapy-sensitive group (Fig. 1A). The expression levels of key glycolytic enzymes, namely GLUT1, ENO1, PGK1, ALDOA, HK1, and LDHA, in the tumor tissues of chemotherapy-resistant and chemotherapy-sensitive groups were examined using IHC analysis. The H-scores of the key glycolytic enzymes in the chemo-resistant group were significantly higher than those in the chemo-sensitive group (Fig. 1B; Supplementary Fig. 1A). The results of western blotting analysis were consistent with those of IHC analysis (Fig. 1C and D). We previously demonstrated that the pseudogene CTSLP8 can regulate the expression of CTSL1 in ovarian cancer through a ceRNA mechanism (Wang et al. 2021). Furthermore, the expression of CTSLP8 was upregulated in patients with DDP-resistant OC in the GEO dataset (Fig. 1E). And the expression of CTSLP8 in OC patients with FIGO III–IV stages was higher than those in OC patients with FIGO I–II stages (Supplementary Fig. 1B). Moreover, the expressions of CTSLP8 among some tumors were also analyzed, which showed the specificity of CTSLP8 in ovarian cancer (Supplementary Fig. 1C). And the upregulated expression of CTSLP8 was associated with poor prognosis in patients with OC (Fig. 1F), and similar trends were observed in other tumors, such as cervical squamous cell carcinoma and endocervical adenocarcinoma (CESC), but the *p* values were not statistically significant (Supplementary Fig. 1D–F). And the RT-PCR analysis on our own collected samples also confirmed that the expression of CTSLP8 was upregulated in the chemotherapy-resistant group (Fig. 1G).

### CTSLP8 promoted proliferation, glycolysis, and DDP resistance in the OC cells

**Fig. 2:**
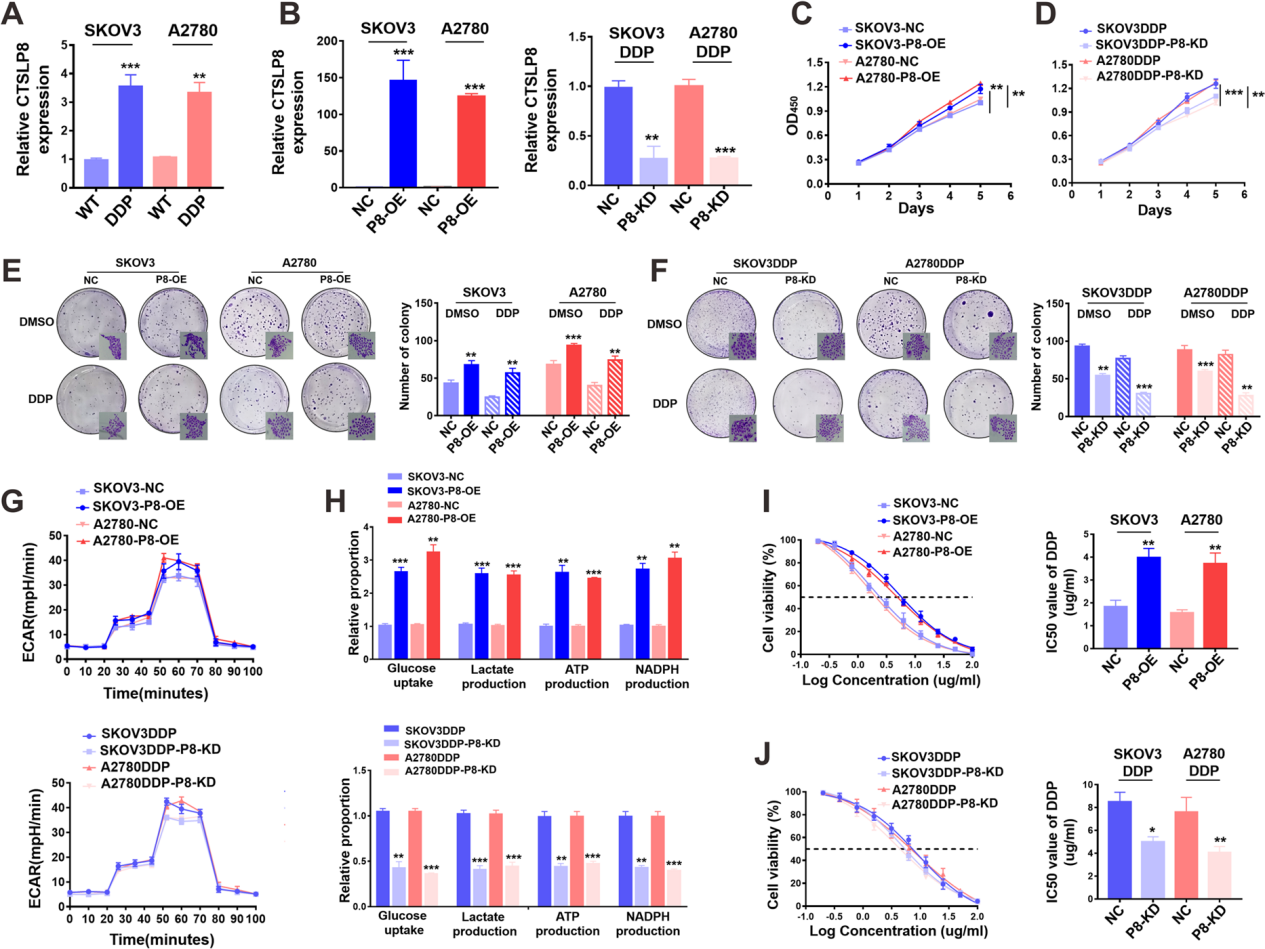
CTSLP8 promoted proliferation, glycolysis, and DDP resistance in OC cells. **A** RT-PCR showed the expression levels of CTSLP8 in four OC cell lines. **B** RT-PCR verified the effects of lentiviruses transfection with CTSLP8 overexpression (OE) or knockdown (KD). **C–D** CCK8 showed the effects of CTSLP8-OE (P8-OE) or CTSLP8-KD (P8-KD) on the proliferation of OC cell lines. **E–F** The clone formation showed the effects of P8-OE or P8-KD on the proliferation of OC cell lines. **G** ECAR assay showed the effects of P8-OE or P8-KD on the glycolysis of OC cell lines. **H** The detection of glycolytic products confirmed the effects of P8-OE or P8-KD on the glycolysis of OC cell lines. **I–J** IC_50_ assay validated the effect of P8-OE or P8-KD on cisplatin resistance of OC cells

The qRT-PCR revealed that the expression levels of CTSLP8 in the SKOV3/SKOV3-DDP and A2780/A2780-DDP cell lines and the results showed that the expression of CTSLP8 in cisplatin-resistant cell lines was higher than that in parental cell lines (Fig. 2A). SKOV3 and A2780 cell lines were transfected with P8-OE constructs, whereas the SKOV3-DDP and A2780-DDP cell lines were treated with P8-KD constructs. The transfection efficiencies of P8-OE and P8-KD were verified using qRT-PCR (Fig. 2B). The results of the CCK8 assay revealed that transfection with P8-OE promoted the proliferation of OC cells, whereas transfection with P8-KD inhibited the proliferation of DDP-resistant OC cell lines (Fig. 2C and D). Similarly, the colony formation assay also revealed that the P8-OE-transfected OC cells exhibited enhanced proliferation, while the P8-KD-transfected DDP-resistant OC cells exhibited decreased proliferation (Fig. 2E and F). The measurement of ECAR revealed that transfection with P8-OE promoted glycolysis in OC cells, whereas transfection with P8-KD inhibited glycolysis in DDP-resistant OC cells (Fig. 2G). The analysis of glycolytic products also confirmed that CTSLP8 upregulated glycolysis in OC cell lines and the results were consistent with those of the ECAR assay (Fig. 2H). The P8-OE-transfected OC cells exhibited increased tolerance to cisplatin, whereas P8-KD-transfected DDP-resistant OC cells exhibited increased sensitivity to cisplatin (Fig. 2I and J).

### CTSLP8 mediated cisplatin resistance in OC through promoting glycolysis

**Fig. 3:**
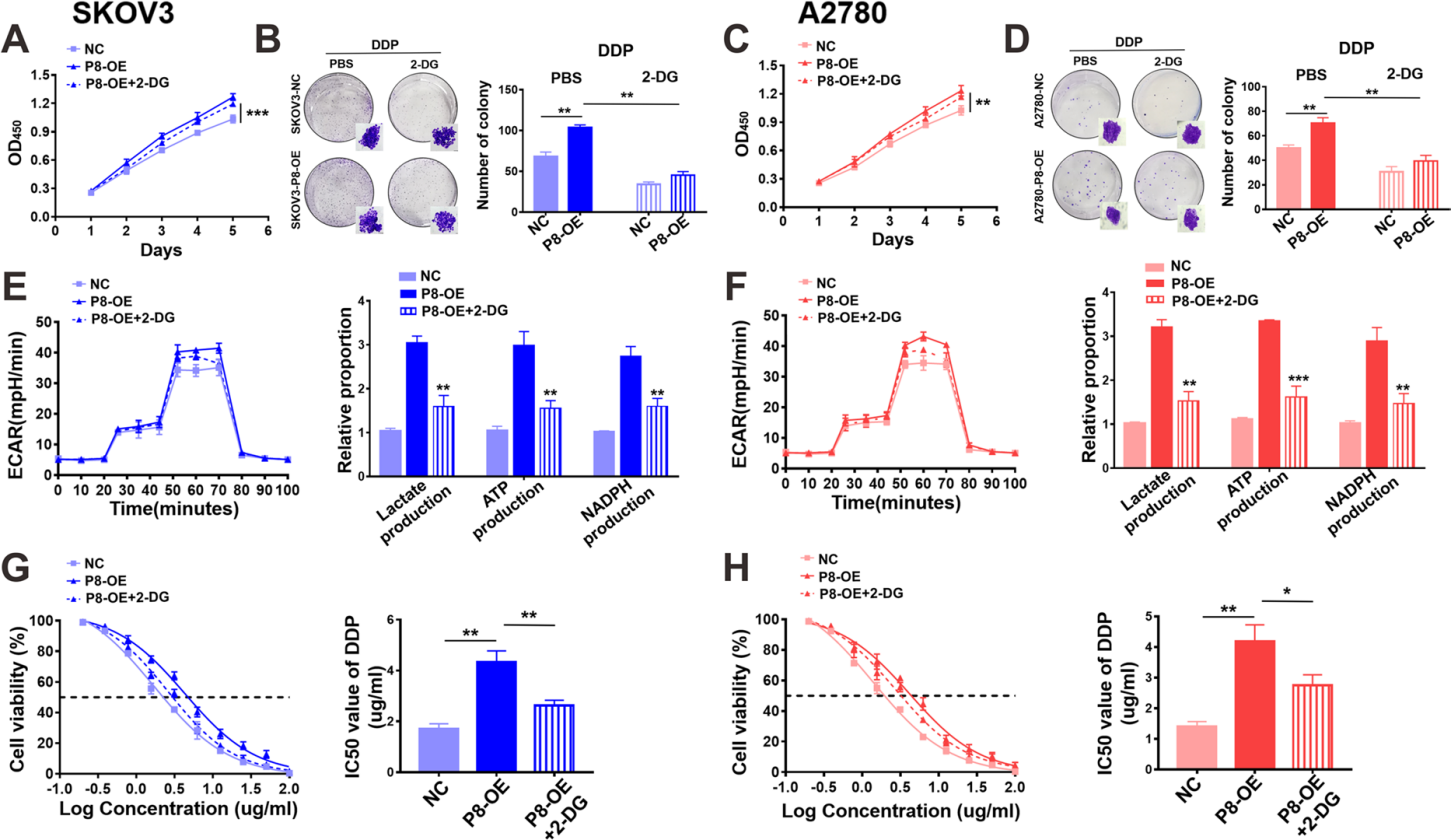
CTSLP8 mediated cisplatin resistance of OC by promoting glycolysis. **A–B** CCK8 and clone formation assay showed the effect of interfering metabolism on proliferation based on SKOV3-P8-OE. **C–D** CCK8 and clone formation assay showed the effect of interfering metabolism on proliferation of A2780-P8-OE. **E–F** ECAR assay and the detection of glycolytic products showed the effects of interfering metabolism on glycolysis of SKOV3-P8-OE/A2780-P8-OE. **G–H** IC_50_ assay validated the effects of interfering metabolism on cisplatin resistance of SKOV3-P8-OE/A2780-P8-OE

The NC-transfected and P8-OE-transfected SKOV3 cells were used for metabolomic analysis. KEGG analysis revealed that the overexpression of CTSLP8 affected the central carbon metabolism in OC cells (Supplementary Fig. 2A). Glycolysis in P8-OE-transfected cells was inhibited by 2-DG. Treatment with 2-DG mitigated the P8-OE-induced enhanced proliferation of SKOV3 cells (Fig. 3A). The results of the colony formation assay concurred with this finding (Fig. 3B). The same phenomenon was observed in the A2780 cell line (Fig. 3C and D). Furthermore, 2-DG mitigated P8-OE-induced enhanced ECAR and glycolysis in OC cells (Fig. 3E and F). Treatment with 2-DG mitigated the P8-OE-mediated enhanced tolerance of OC cells to cisplatin (Fig. 3G and H).

### CTSLP8 directly bound PKM2 and positively regulated c-Myc expression

**Fig. 4:**
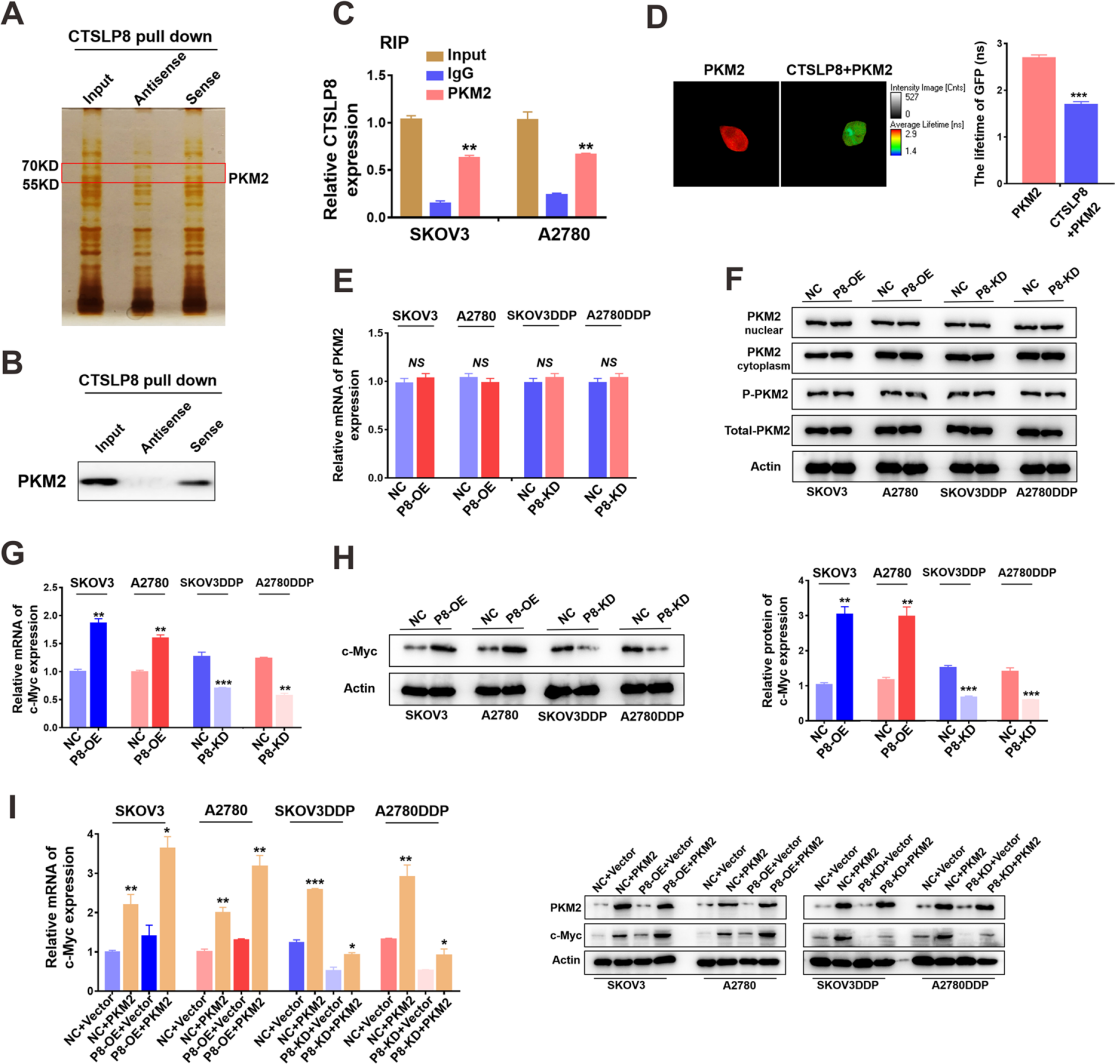
CTSLP8 bound to PKM2 directly and positively correlated with c-Myc expression. **A** RNA pull-down combined with mass spectrometry showed the proteins binding directly to CTSLP8. **B** RNA pull-down combined with WB showed that CTSLP8 binds to PKM2 directly. **C** RIP assays verified that PKM2 binds to CTSLP8. **D** FRET confirmed that CTSLP8 interacted with PKM2. **E–F** The changes in levels of CTSLP8 neither affected the expression of PKM2 in mRNA level nor the protein level. **G–H** RT-PCR and western blot showed the expression of c-Myc in OC cell lines with different CTSLP8 expressions. **I** RT-PCR and western blot showed that upregulation of PKM2 expression promoted the expression of c-Myc in P8-NC/P8-OE cell lines

The results of the RNA pull-down and mass spectrometry revealed the proteins to binding directly to CTSLP8. Of these, CTSLP8 specifically bound to PKM2 (Fig. 4A). RNA pull-down and western blotting analyses revealed that CTSLP8 directly binds to PKM2 (Fig. 4B). Consistently, the results of the RIP assays revealed the binding between PKM2 and CTSLP8 (Fig. 4C). The PKM2-GFP and CTSLP8-RFP plasmids were co-transferred into the SKOV3 cells (Supplementary Fig. 2B). FRET analysis revealed that the lifetime of GFP was shorter than the reported level, which confirmed that CTSLP8 interacted with PKM2 (Fig. 4D). This indicates that CTSLP8 directly binds to PKM2. However, changes in the expression level of CTSLP8 had no effect on the mRNA or protein expression of PKM2 (Fig. 4E and F).

C-Myc is a vital transcription factor involved in both tumor metabolism and drug resistance. The qRT-PCR and western blotting analyses revealed that the expression of c-Myc in OC cell lines was proportional to that of CTSLP8 (Fig. 4G and H). Next, the PKM2 overexpression plasmid was transfected into P8-OE-transfected or P8-KD-transfected OC cells. The overexpression of PKM2 was confirmed at the mRNA and protein levels (Supplementary Fig. 2C and D). The qRT-PCR and western blotting analyses revealed that the expression of c-Myc increased in PKM2-overexpressing cells, especially in the P8-OE-transfected cell lines (Fig. 4I; Supplementary Fig. 2E). Therefore, CTSLP8 was hypothesized to promote the regulation of c-Myc by PKM2.

### CTSLP8 and PKM2 formed transcription complexes and regulated the expression of c-Myc

**Fig. 5:**
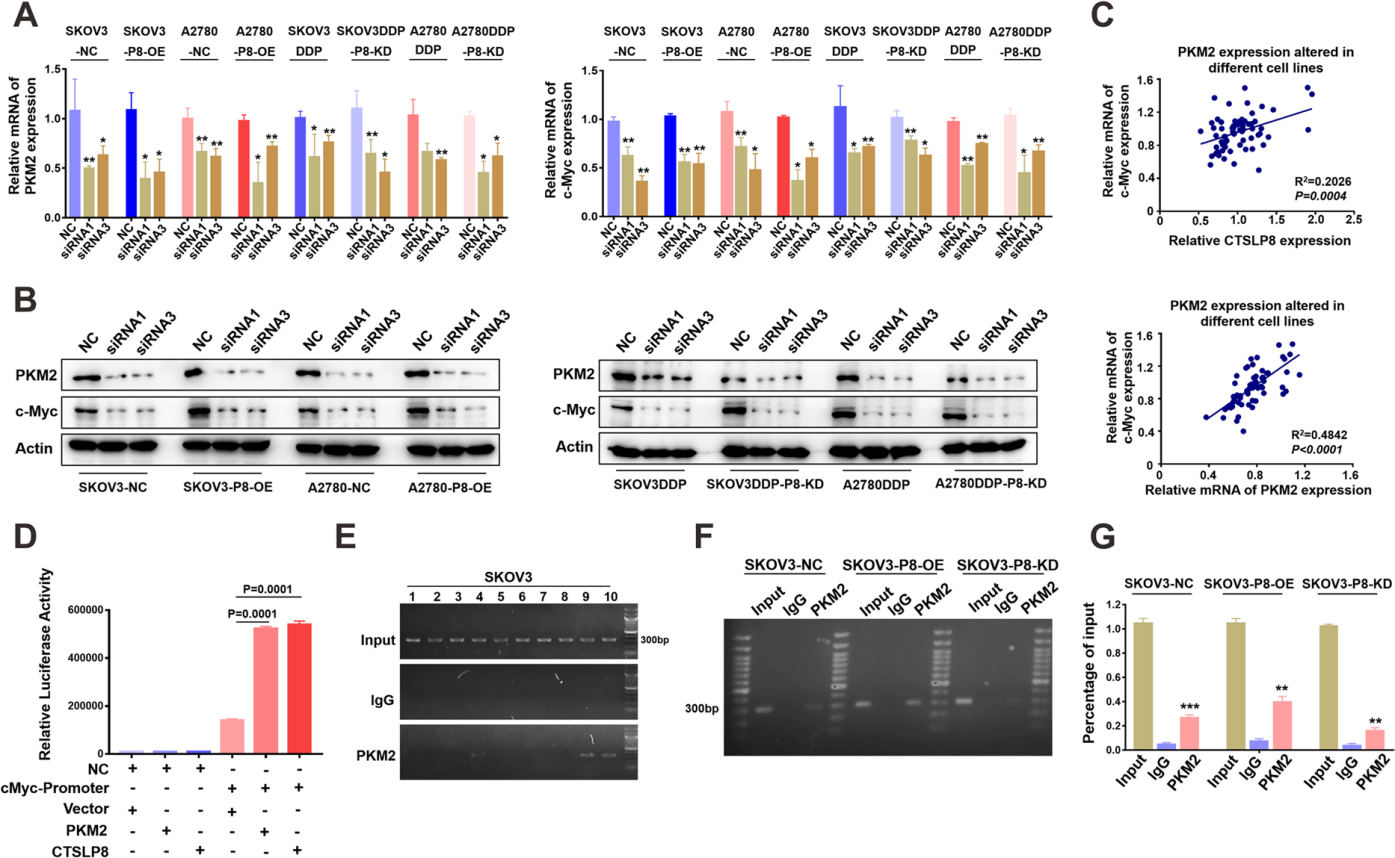
Lnc-CTSLP8 and PKM2 formed transcription complexes and regulated the expression of c-Myc. **A** RT-PCR showed the effect of PKM2 knockdown on c-Myc in OC cell lines with different CTSLP8 expressions. **B** Western blot showed the effect of PKM2 knockdown on c-Myc in OC cell lines with different CTSLP8 expression. **C** The correlation between c-Myc and PKM2 or c-Myc and CTSLP8 expression when PKM2 expression was disturbed by siRNAs. **D** The dual-luciferase reporter assay confirmed that PKM2 or CTSLP8 could bind to c-Myc promoter. **E** The ChIP assay confirmed that PKM2 binds to c-Myc promoter. **F–G** The ChIP assay showed the binding of PKM2 to c-Myc promoter in cell lines with different CTSLP8 expressions

The siRNAs targeting PKM2 were transfected into P8-OE-transfected or P8-KD-transfected OC cells. The knockdown efficiency of PKM2 siRNAs was verified at the mRNA and protein levels. The siRNA1 and siRNA3 of PKM2 were more effective in knocking down PKM2, and they were used for the experiments (Supplementary Fig. 2F and G). The qRT-PCR analysis revealed that the knockdown of PKM2 downregulated the expression of c-Myc in both P8-OE-transfected and P8-KD-transfected cell lines (Fig. 5A). The results of qRT-PCR analysis were consistent with those of western blot analysis (Fig. 5B; Supplementary Fig. 2H). Then, results from different cell lines were summarized, and the mRNA expression level of PKM2 and CTSLP8 was positively correlated with c-Myc expression (Fig. 5C). Moreover, immunofluorescence analysis also revealed that the expression of PKM2 was also directly proportional to c-Myc expression in SKOV3-P8-OE cells (Supplementary Fig. 2I).

Bioinformatics analysis predicted that PKM2 binds to the c-Myc promoter region (Supplementary Fig. 3A and B). Moreover, the dual-luciferase reporter assay confirmed that PKM2 and CTSLP8 could bind to the promoter region of c-Myc (Fig. 5D). The results of the ChIP assay confirmed that PKM2 binds to the promoter region of c-Myc (Fig. 5E; Supplementary Fig. 3C). The results of the ChIP assay also confirmed that the overexpression of CTSLP8 promoted the binding of PKM2 to the c-Myc promoter (Fig. 5F and G).

### CTSLP8 promoted OC progression and drug resistance in vivo

**Fig. 6:**
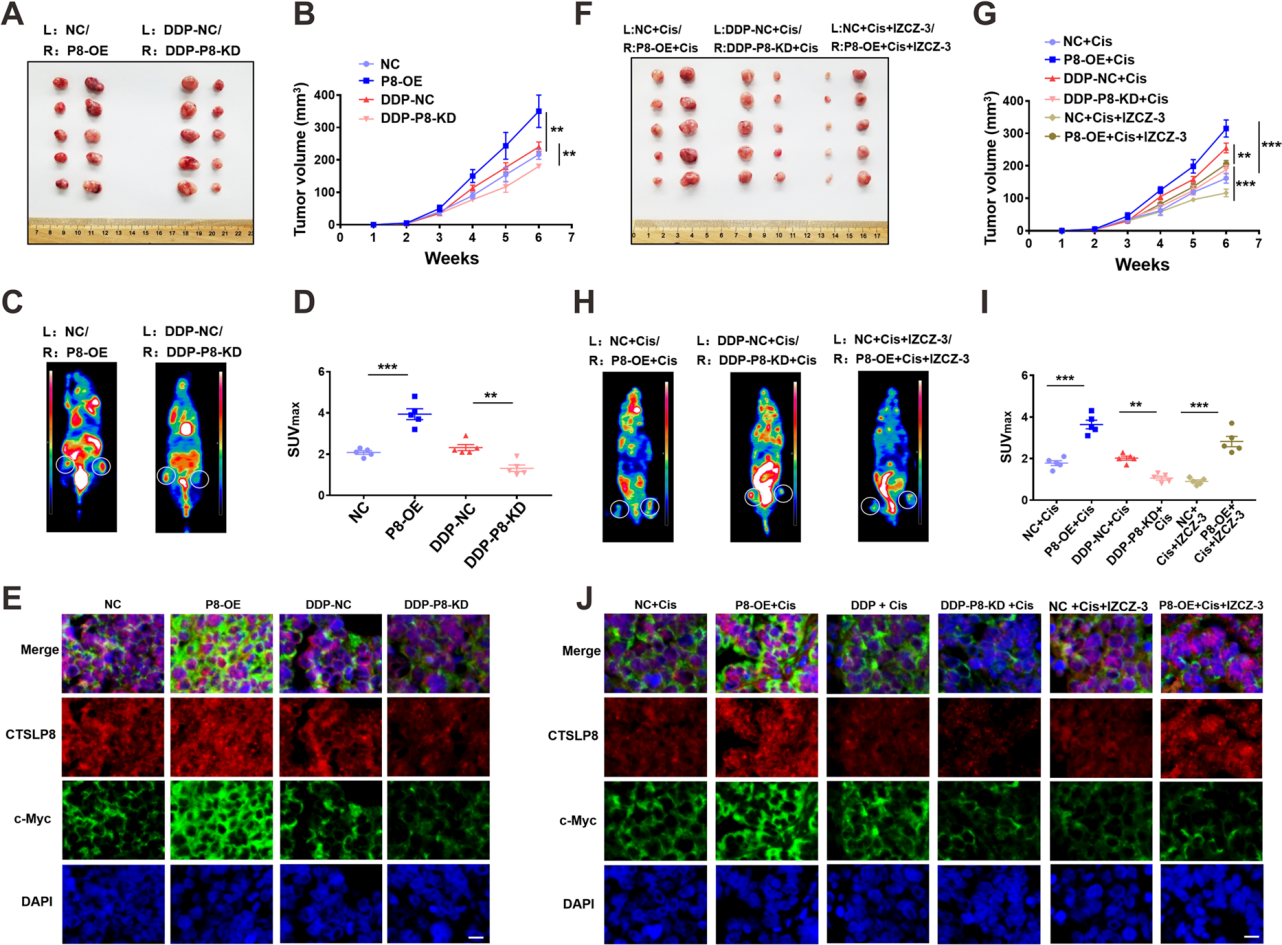
CTSLP8 was associated with the progression and drug resistance of OC in vivo. **A** Anatomic diagram about subcutaneous injection of SKOV3 cells with different CTSLP8 expressions at week 6. L, left; R, right. **B** Tumor volumes after subcutaneous injection of SKOV3 cells treated differently. **C** PET-CT with subcutaneous injection of SKOV3 cells with different CTSLP8 expression at week 6. **D** SUV maximum value of tumor in different groups. **E** Immunofluorescence showed the expression of c-Myc and CTSLP8 in different group. Scale bar, 10 µm. **F** Anatomic diagram about subcutaneous injection of SKOV3 cells with different drug treatments at week 6. L, left; R, right; Cis, cisplatin. **G** Tumor volumes after subcutaneous injection of SKOV3 cells treated differently. **H** PET-CT with subcutaneous injection of SKOV3 cells in all treatments at week 6. **I** SUV maximum value of tumor in all treatments. **J** Immunofluorescence showed the expression of c-Myc and CTSLP8 in all treatments. Scale bar, 10 µm

P8-OE-transfected and P8-KD-transfected SKOV3 cells were transplanted on both sides of nude mice for comparison to minimize individual differences. NC cells were subcutaneously injected into the left side, while the P8-OE-transfected or P8-KD-transfected cells were subcutaneously injected into the right side. The image of the tumor at week 6 post-injection was present (Fig. 6A). Additionally, tumor growth was rapid in the CTSLP8-high expression group (Fig. 6B). The PET-CT scan revealed that subcutaneous tumors with high CTSLP8 expression exhibited high metabolism and SUV values at week 6 post-injection (Fig. 6C). And the SUV values in different groups were positively correlated with the expression of CTSLP8 (Fig. 6D). Immunofluorescence analysis revealed that the expression of c-Myc was upregulated in the tumor tissues exhibiting enhanced CTSLP8 expression (Fig. 6E). By IHC staining, it was found that the expression of glycolytic enzymes was positively correlated with that of CTSLP8 and c-Myc (Supplementary Fig. 3D).

Nude mice were also subcutaneously injected with NC-transfected SKOV3 cells into the left side and P8-OE-transfected or P8-KD-transfected SKOV3 cells into the right side, followed by injection with cisplatin from the second week. The image of the tumor at week 6 was present (Fig. 6F). Consistently, this combination decreased the tumor volume (Fig. 6G). The SUV values obtained from the PET-CT scan revealed that the combination of cisplatin and c-Myc inhibitor (IZCZ-3) exerted the most potent inhibitory effect on tumor metabolism (Figs. 6H-I). Immunofluorescence analysis revealed that the combination of cisplatin and c-Myc inhibitor (IZCZ-3) inhibited the expression of c-Myc (Fig. 6J). IHC staining revealed that the expression of glycolytic enzymes was positively correlated with that of CTSLP8 and c-Myc (Supplementary Fig. 3E).

### Upregulated CTSLP8 expression in the OC tissues was associated with chemotherapy resistance and poor prognosis

**Fig. 7:**
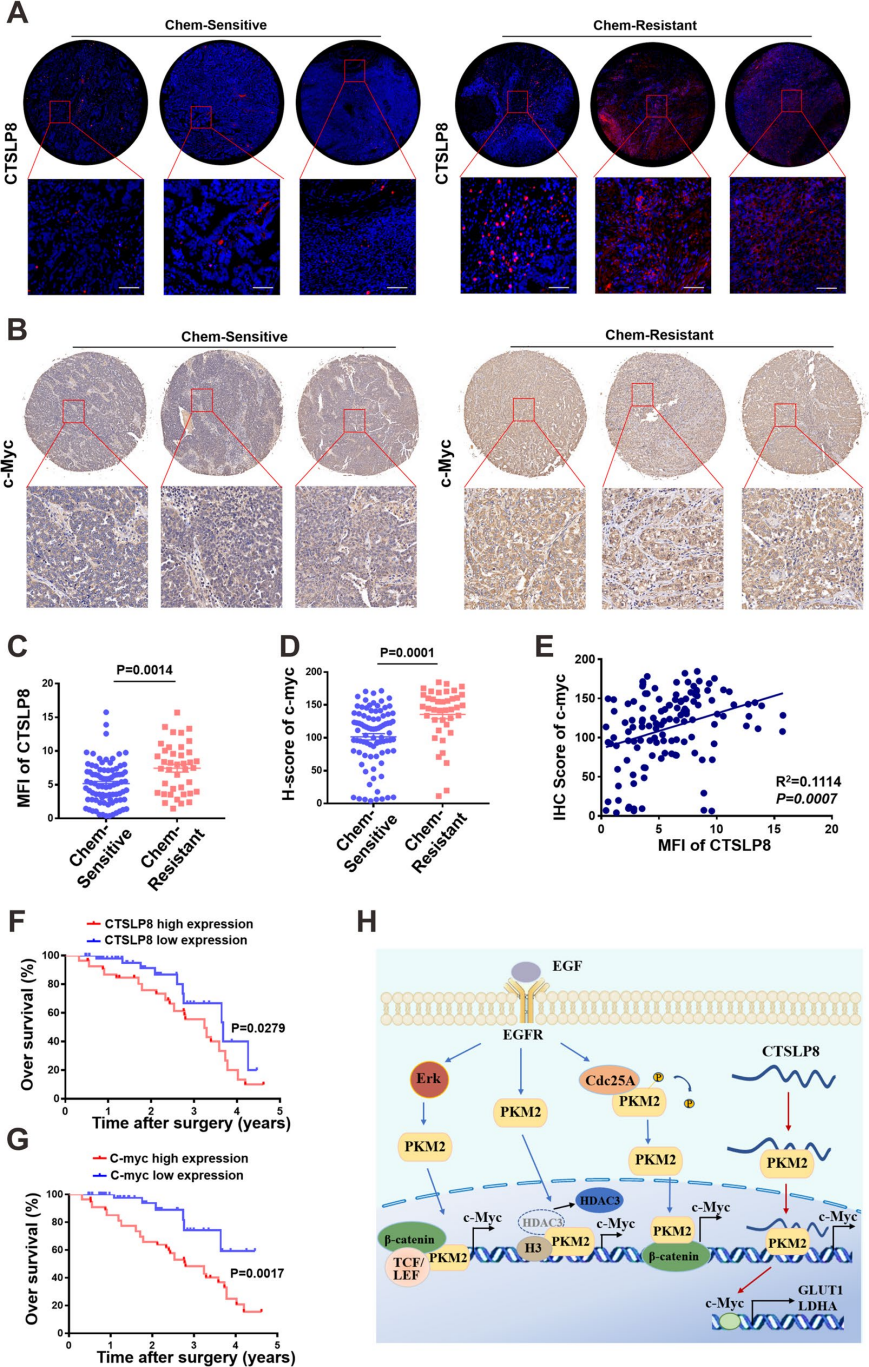
High expression of CTSLP8 in OC tissues was associated with chemotherapy response and overall survival. **A** In situ hybridization experiments demonstrated the expression of CTSLP8 with different chemotherapy response on tissue chips. **B** Representative immunohistochemistry staining demonstrated the expression of the c-Myc with different chemotherapy response on tissue chips. **C–D** The mean fluorescence intensity (MFI) and H-score were used to quantify the expressions of CTSLP8 and c-Myc, respectively. **E** The correlation between mean fluorescence intensity (MFI) of CTSLP8 and H-score of c-Myc. **F** Patients with OC with a high CTSLP8 expression exhibited a significantly low overall survival. **G** Patients with OC with a high c-Myc expression exhibited a significantly low overall survival. **H** Diagram illustrating our conclusions

The tumor chips were subjected to FISH and IHC analyses to examine the expression patterns of CTSLP8, PKM2, and c-Myc. The expression of CTSLP8 was upregulated in the chemotherapy-resistant group (Fig. 7A). Consistently, IHC analysis revealed that c-Myc expression was upregulated in the chemotherapy-resistant group (Fig. 7B). The expressions levels of CTSLP8 and c-Myc were quantified by mean fluorescence intensity (MFI) and H-score, respectively (Fig. 7C and D). The MFI of CTSLP8 and the H-score of c-Myc, which were automatically assessed after scanning, were positively correlated (Fig. 7E). Patients with OC exhibiting upregulated CTSLP8 expression were associated with a significantly low OS (Fig. 7F). Additionally, the prognosis of patients with OC exhibiting upregulated c-Myc expression was also poor (Fig. 7G).

These results indicate that CTSLP8 binds to PKM2 and promotes its binding to the region of c-Myc promoter, which leads to the upregulation of c-Myc expression. The regulation of c-Myc transcription by PKM2 has been reported for many times. EGFR-activated ERK2 binds directly to PKM2 through the ERK2 docking groove, which promotes PKM2 translocating to the nucleus. Then, PKM2 acts as a coactivator of β-catenin to induce c-Myc expression, resulting in the upregulation of GLUT1 and LDHA (Yang, et al. 2012a, b). Furthermore, PKM2 directly binds to histone H3 and phosphorylates histone H3, and this phosphorylation is required for the dissociation of HDAC3 from the c-Myc promoter regions, which induces expression of cyclin D1 and c-Myc, tumor cell proliferation, cell cycle progression, and brain tumorigenesis (Yang, et al. 2012a, b). EGFR activation also results in c-Src-mediated Cdc25A phosphorylation, and Cdc25A can dephosphorylate PKM2, which promotes c-Myc-upregulated expression and then increases the expression of glycolytic GLUT1, PKM2, and LDHA (Liang et al. 2016). We provided an additional figure to explain the molecular mechanisms, where we include mechanisms published by others in blue and the mechanisms found by own in red (Fig. 7H).

## Discussion

Previous studies have reported that the pseudogene CTSLP8 is upregulated in breast cancer and that the upregulated CTSLP8 is an indicator of poor prognosis in breast cancer. However, these studies only involved bioinformatics analysis and did not provide experimental evidence (Smerekanych et al. 2020). Pseudogenes, which exist in almost all cells, are similar to various protein-coding genes. Although pseudogenes lack protein-coding functions, they are involved in various critical biological functions (Cheetham et al. 2020). High-throughput sequencing analysis has identified pseudogenes as gene repositories that store and expand genetic information. Additionally, pseudogenes are involved in the progression of various diseases, especially cancer (Chen et al. 2020). The role of pseudogenes in cancer is complex and requires further study. Previously, we reported that CTSLP8 regulated CTSL1 expression through a ceRNA mechanism as a pseudogene (Wang et al. 2021). In this study, we demonstrated that CTSLP8 expression was upregulated in the chemotherapy-resistant OC tissues and that it promoted proliferation, glycolysis, and DDP resistance in OC cells.

The different isoforms of pyruvate kinase exhibit characteristic expression patterns. In cancer cells, PKM2 is the predominant isoform (Méndez-Lucas et al. 2017). PKM2 plays a critical role in the reprogramming of cell metabolism, which is essential for tumorigenesis. Unlike other metabolic enzymes, PKM2 coexists in multiple forms, such as monomers, dimers, and tetramers. Moreover, the PKM2 dimer could promote aerobic glycolysis through transcriptional regulation (Wong et al. 2015). PKM2 in HCC-derived ectosomes has been reported to induce macrophage differentiation by regulating metabolic reprogramming. Additionally, PKM2 promotes the acetylation of H3K9 and H4K8 histones in the promoter region of key transcription factors. Furthermore, PKM2 activates STAT3 phosphorylation and promotes its binding to the promoter region (Hou et al. 2020). Thus, PKM2 can function as a kinase or a transcriptional regulator. The findings of this study also confirmed that PKM2 bound to the promoter region of c-Myc and regulated its transcription.

The Myc oncogene contributes to tumorigenesis in several human cancers. Myc proteins belong to the nuclear transcription factor family comprising c-Myc, n-Myc, and l-Myc, which regulate cell growth, cell cycle, metabolism, and survival (Dang 2012). c-Myc is demonstrated to mediate drug resistance in cancer. The inhibition of c-Myc is a potential therapeutic strategy for colorectal cancer (Elbadawy et al. 2019). PKM2 and c-Myc are involved in tumor development. For example, the activity and the expression of PKM2 is involved in c-Myc-mediated glucose catabolism and tumorigenesis in liver tumors (Méndez-Lucas et al. 2017). The c-Myc-PKM2 axis, which plays critical role in oncogenic cellular proliferation in head and neck cancer (HNC), promotes glycolysis and chemotherapy resistance in HNC cells (Gupta et al. 2018). The regulation of c-Myc transcription by PKM2 has also been summarized in Fig. 7H, which confirms that PKM2 can regulate the c-Myc promoter region in variety ways, thus affecting the downstream glucose metabolism. As for lncRNAs, the lncRNA/protein complex is reported to mediate the transcriptional activation of c-Myc. The HBXIP/lncRNA Hotair/LSD1 complex promotes the c-Myc-mediated transcription of cyclin A, eIF4E, and LDHA in the breast cancer cells (Li et al. 2016). In this study, CTSLP8 was found directly bind to PKM2. However, the expression of PKM2 was not affected. The CTSLP8/PKM2 complex upregulated the expression of c-Myc by binding to the promoter region, which resulted in the upregulation of glycolysis and the development of DDP resistance in OC.

The inhibition of c-Myc oncogene is a potential strategy for cancer treatment. C-Myc inhibitors are classified based on their mechanism of action, including inhibiting the interaction between Max and c-Myc, modulating the transcriptional activity of c-Myc, and promoting the degradation of c-Myc. For example, stauprimide can combine with NME2 and interfere with its nuclear localization in embryonic stem cells (ESCs), while NME2 is an important transcription factor of Myc. Previous studies have reported that stauprimide increases the efficiency of differentiation of mouse and human ESCs through the downregulation of c-Myc (Zhu et al. 2009). Stauprimide selectively suppresses Myc transcription in different cancer cell lines and inhibits tumor progression in xenograft models (Bouvard et al. 2017). IZCZ-3 blocks the G-quadruplex structure on the promoter region of c-Myc, which interferes with c-Myc transcription and leads to apoptosis, cell cycle arrest, and cell growth inhibition. Additionally, IZCZ-3 effectively suppresses tumor progression in xenograft model (Hu et al. 2018). Therefore, IZCZ-3 was used as a c-Myc inhibitor in this study to treat subcutaneous tumors.

In conclusion, the findings of this study indicated that CTSLP8 could directly bind to PKM2 and that the CTSLP8/PKM2 complex upregulated c-Myc expression by interacting with the promoter region. This resulted in enhanced proliferation and glycolysis and the development of DDP resistance in OC cells. The downregulation of CTSLP8 and the application of chemotherapeutic drugs in combination with c-Myc inhibitors were potential therapeutic strategies for patients with chemotherapy-resistant OC.

## Supplementary Information

Below is the link to the electronic supplementary material.

Supplementary file1 (DOCX 1518 KB)

## Data Availability

The datasets used and/or analyzed during the current study are available from the corresponding author on reasonable request.
