# Supplementary material for: Long non-coding RNA CTSLP8 mediates ovarian cancer progression and chemotherapy resistance by modulating cellular glycolysis and regulating c-Myc expression through PKM2
Source: Cell Biol Toxicol. 2021 Sep 12;38(6):1027–45. doi: 10.1007/s10565-021-09650-9 (PMC9750935; doi:10.1007/s10565-021-09650-9)
Supplement: Supplementary file 1 — Supplementary file1 (DOCX 1518 KB) [file 10565_2021_9650_MOESM1_ESM.docx]

**Supplement data**


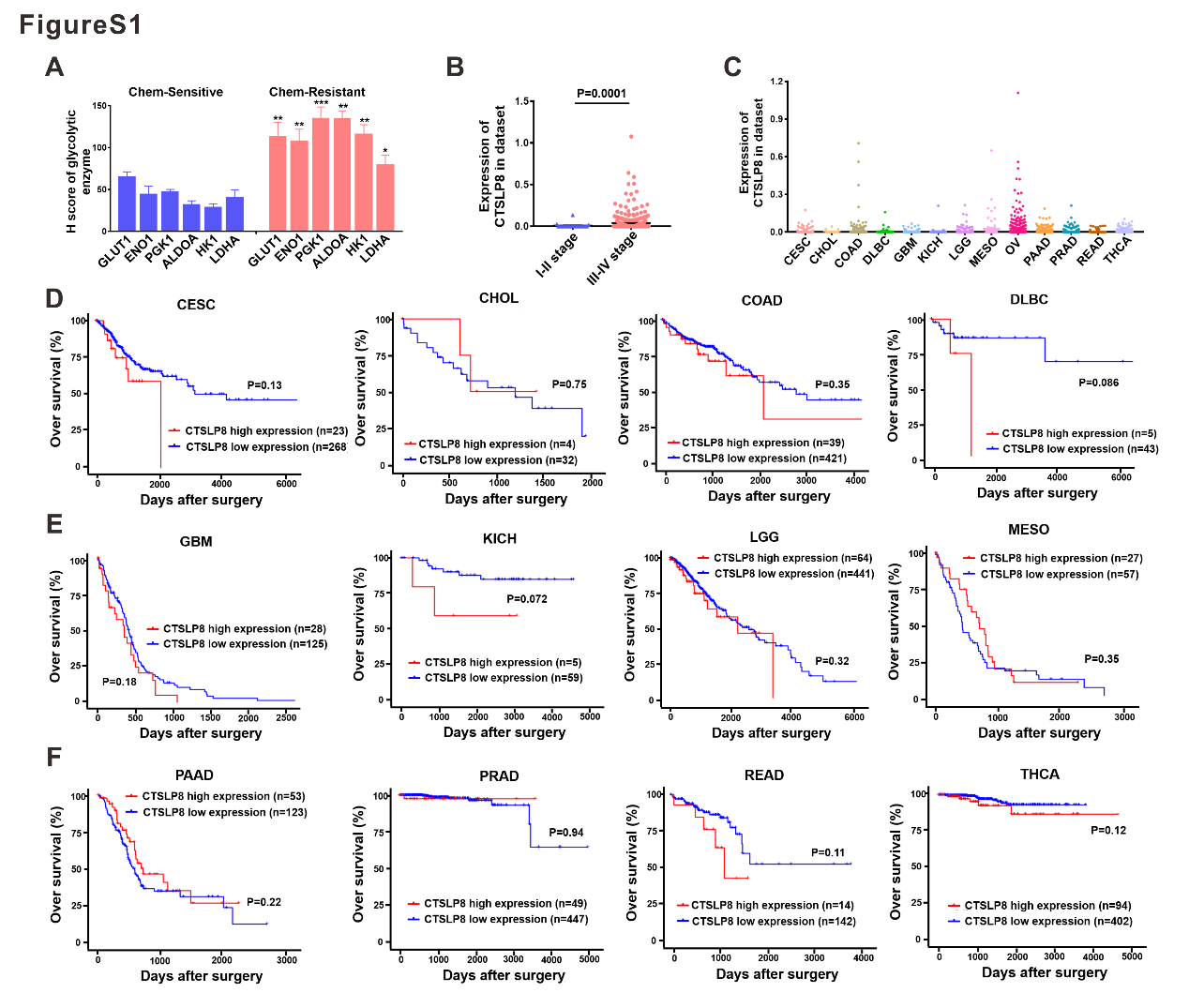


**Figure S1. CTSLP8 was specifically high expressed in OC patients.**

(**A**) H-score of the key glycolytic enzymes in the chemo-resistant group and chemo-sensitive group. **(B)** The expression of CTSLP8 in OC patients with FIGO I-II stage and FIGO III-IV stage OC patients from TCGA dataset. **(C)** The expressions of CTSLP8 among some tumors. (**D-F**) The OS of tumor patients in different CTSLP8 expression groups from dataset. CESC, Cervical squamous cell carcinoma and endocervical adenocarcinoma. CHOL, Cholangiocarcinoma. COAD, Colon adenocarcinoma. DLBC, Lymphoid Neoplasm Diffuse Large B-cell Lymphoma. GBM, Glioblastoma multiforme. KICH, Kidney Chromophobe. LGG, Brain Lower Grade Glioma. MESO, Mesothelioma. OV, Ovarian serous cystadenocarcinoma. PAAD, Pancreatic adenocarcinoma. PRAD, Prostate adenocarcinoma. READ, Rectum adenocarcinoma. THCA, Thyroid carcinoma


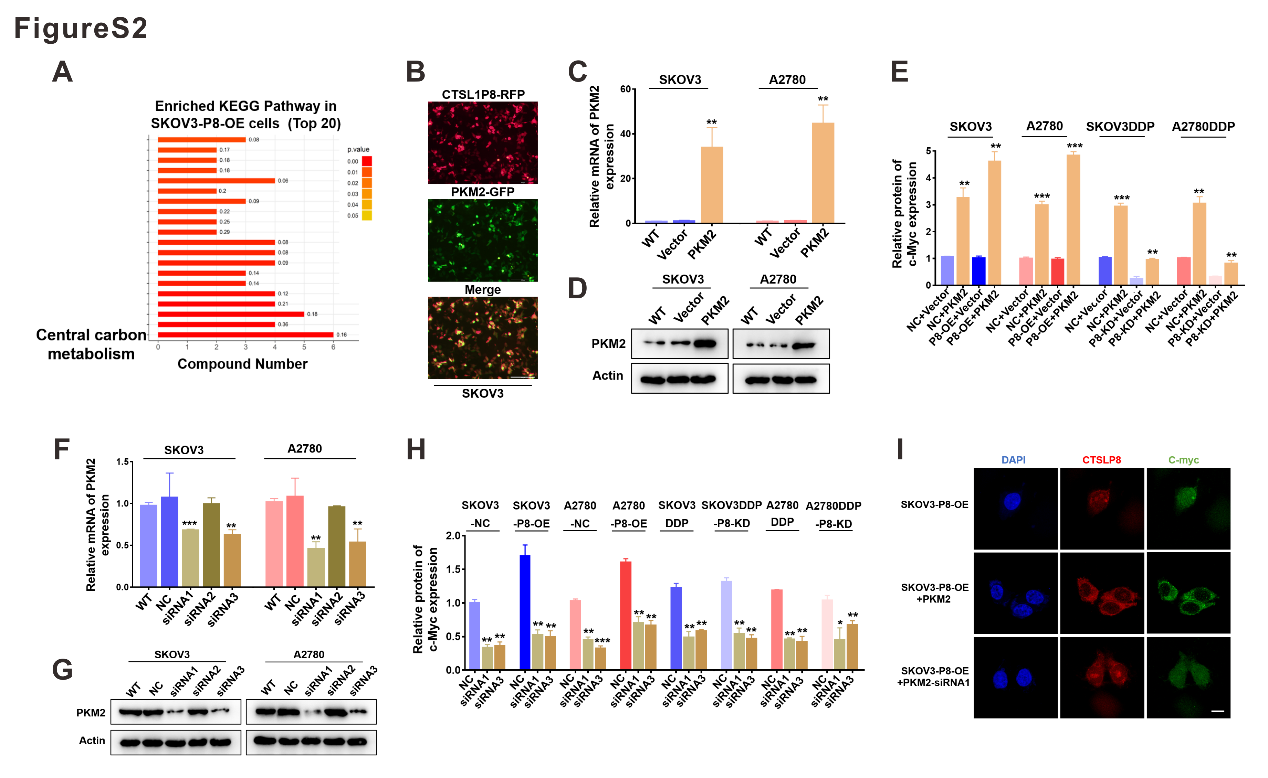


**Figure S2. The expression of CTSLP8 and PKM2 were positively correlated with the expression of c-Myc.**

**(A)** Enriched KEGG Pathway in metabolomics showed that the central carbon metabolism was most affected after CTSLP8 over-expression. (**B**) SKOV3 cells was co-transfected with PKM2-GFP plasmid and CTSLP8-RFP plasmid after 36h. Scale bar, 200 µm. **(C-D)** The overexpression efficiency of PKM2 plasmid was demonstrated at both protein and RNA levels. **(E)** The gray values showed the expression of c-Myc after PKM2 overexpression in different CTSLP8 expression cell lines. (**F-G**) The knockdown efficiency of PKM2 siRNAs was demonstrated at both protein and RNA levels. (**H**) The gray values showed the expression of c-Myc while PKM2 was inhibited by siRNAs. **(I)** Immunofluorescence showed CTSLP8 expression was positively correlated with c-Myc expression. Scale bar, 10 µm.


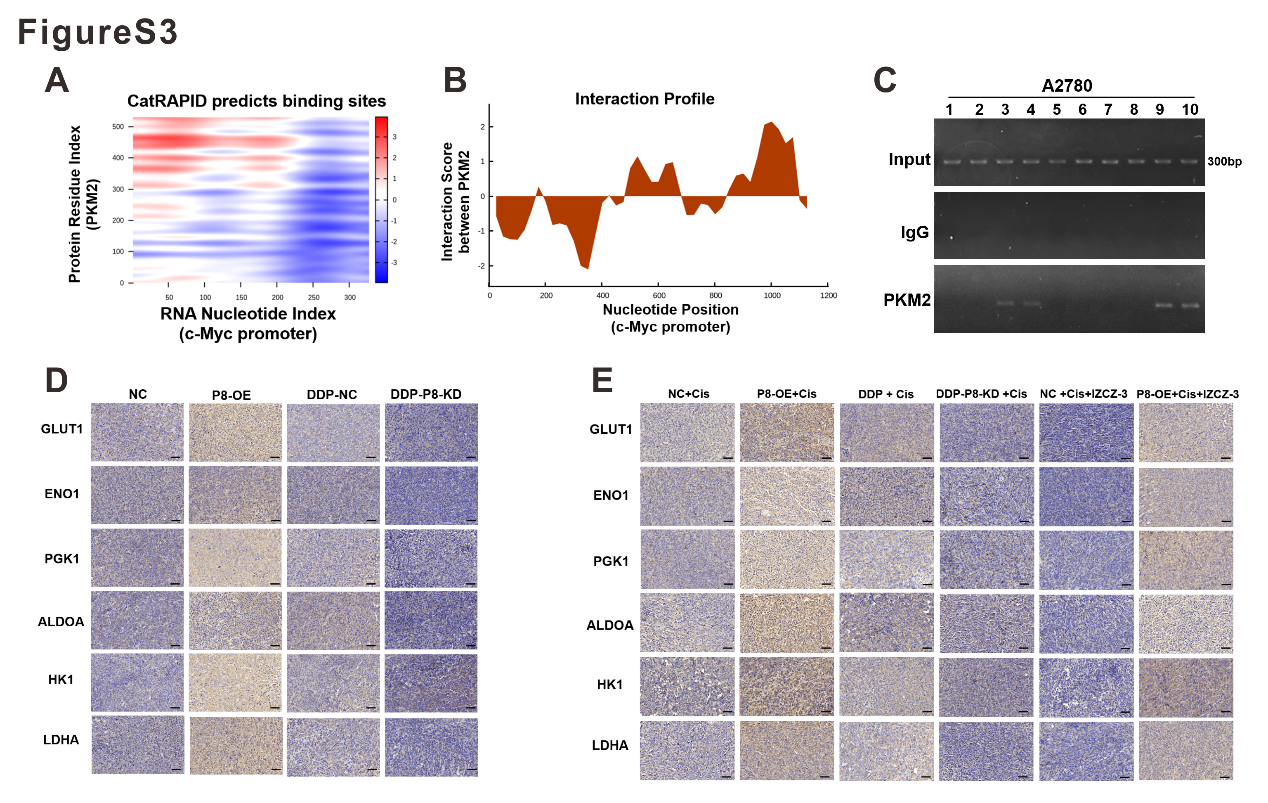


**Figure S3. CTSLP8 and PKM2 regulated glycolysis of OC through c-Myc.**

(**A-B**) Bioinformatics analysis predicted that PKM2 could bind to the c-Myc promoter region. (**C**) The ChIP assay confirmed that PKM2 bound to c-Myc promoter in A2780 cells. (**E-F**) IHC staining of glycolytic enzymes performed on tumor tissues in different groups. Scale bar, 50 µm.
